# Catalytic RNA Oligomers Formed by Co-Oligomerization of a Pair of Bimolecular RNase P Ribozymes

**DOI:** 10.3390/molecules27238298

**Published:** 2022-11-28

**Authors:** Mst. Ayesha Siddika, Takahiro Yamada, Risako Aoyama, Kumi Hidaka, Hiroshi Sugiyama, Masayuki Endo, Shigeyoshi Matsumura, Yoshiya Ikawa

**Affiliations:** 1 Graduate School of Innovative Life Science, University of Toyama, Gofuku 3190, Toyama 930-8555, Japan; 2 Department of Chemistry, Graduate School of Science and Engineering, University of Toyama, Gofuku 3190, Toyama 930-8555, Japan; 3 Institute for Integrated Cell-Material Sciences, Kyoto University, Kyoto 606-8501, Japan; 4 Organization for Research and Development of Innovative Science and Technology, Kansai University, Osaka 564-8680, Japan

**Keywords:** ribonuclease P, ribozyme, RNA motif, RNA nanostructure, pre-tRNA processing

## Abstract

Naturally occurring ribozymes with a modular architecture are promising platforms for construction of RNA nanostructures because modular redesign enables their oligomerization. The resulting RNA nanostructures can exhibit the catalytic function of the parent ribozyme in an assembly dependent manner. In this study, we designed and constructed open-form oligomers of a bimolecular form of an RNase P ribozyme. The ribozyme oligomers were analyzed biochemically and by atomic force microscopy (AFM).

## 1. Introduction

Proteins and RNAs are biopolymers with the ability to fold into a variety of three-dimensional tertiary structures and to exhibit biological functions [1,2]. Beyond the self-folding ability of monomeric proteins, some proteins can assemble into higher-order quaternary complexes, in which the respective protein molecules act as subunits [3,4]. Protein quaternary complexes often exhibit sophisticated functions that cannot be achieved by their monomeric homologues lacking assembly ability. Protein quaternary complexes exhibit structural diversity depending on the number of subunits and extent of symmetry in their assembly. The physical stability and rigidity of the assembly states are also highly diverse. Some exhibit liquid-like properties when protein assembly is highly dynamic and metastable [5,6]. Ordered quaternary complexes with high structural stability can also be formed through symmetrical assembly to build geometric shapes, such as triangular trimers (e.g., λ exonuclease), hexagonal hexamers (e.g., T7 gp4 helicase), and oligomers with polyhedral shapes (e.g., virus capsids) [3].

The structures and properties of self-folding RNAs share similarity with proteins in their monomeric states, and RNA molecules can also form quaternary complexes with protein. These RNA–protein complexes exhibit various structural and biophysical properties ranging from liquid-like properties [7,8] to nanoscale machinery, such as the ribosome and spliceosome [9,10,11,12,13,14]. By comparison between protein-based and RNA-based quaternary complexes, we found occasional oligomeric assembly of structured RNAs without protein components [15]. One exceptional example is pRNA of bacteriophage phi29, which forms polygonal-shaped oligomeric states through intermolecular noncovalent interactions even without protein components [15,16,17].

To complement the limited number of naturally occurring RNAs that assemble with geometrical shapes, we have developed artificial RNA assemblies by using modular group I ribozymes [18,19,20,21,22]. We reorganized the catalytic module and structural module composing the *Tetrahymena* group I ribozyme to achieve intermolecular assembly [18,19]. The resulting variant RNAs selectively formed module–module interactions exclusively in an intermolecular manner to form oligomeric states. Depending on the relative positions of the activator and catalytic modules, variant ribozymes formed either open-form [19] or closed-form oligomers with polygonal shapes [18,20].

**Figure 1:**
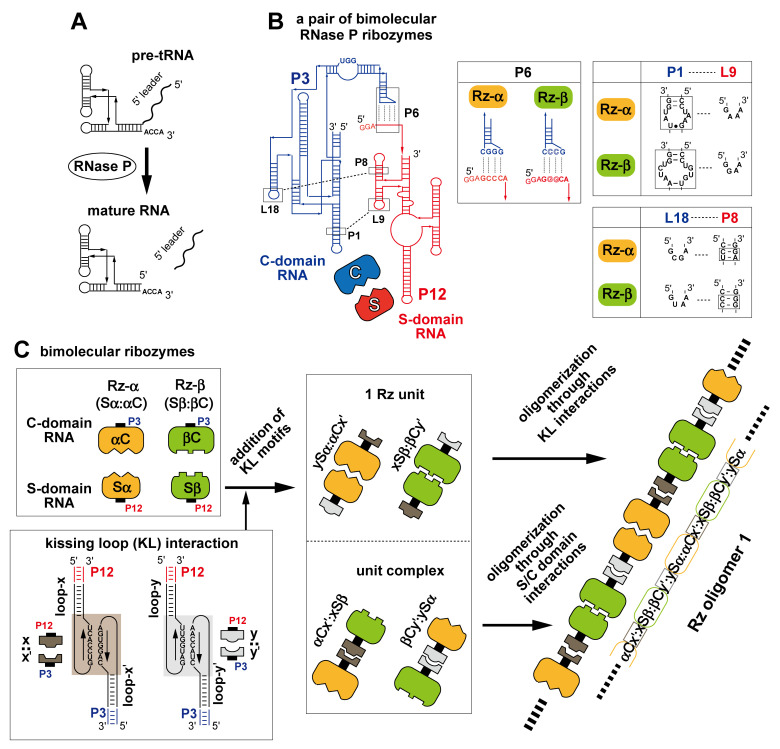
Reaction, modular structure, and assembly of RNase P ribozymes. (**A**) Removal of the 5′ leader sequence of pre-tRNA catalyzed by RNase P enzymes. (**B**) Secondary structure of a bimolecular bacterial RNase P ribozyme consisting of the S-domain RNA (shown in red) and C-domain RNA (shown in blue). Broken lines indicate tertiary structures participating in noncovalent assembly between the C-domain and S-domain. Assembly of the two-domain RNAs was mediated by a trio of tertiary interactions (P1–L9, L18–P8, and P6), by which RNA–RNA assembly interfaces between the C-domain and S-domain were defined and supported. (**C**) Scheme of modular engineering to generate bimolecular RNase P ribozymes possessing KL motifs enabling co-oligomerization to yield Rz oligomer 1.

Artificial construction of a series of oligomeric structures based on the *Tetrahymena* ribozyme demonstrated that modular ribozymes are promising platforms to generate RNA nanostructures with catalytic properties. Bacterial ribonuclease P ribozymes, which cleave precursor tRNAs (pre-tRNAs) to remove their 5′ leader sequences (Figure 1A), also represent a promising class of modular ribozymes because of their modular architecture [23,24,25]. RNase P ribozymes consist of two structural modules, the S-domain and C-domain, which can be dissected physically and can fold independently without the partner module (Figure 1B) [26,27,28,29]. These two-domain RNAs reconstitute the active ribozyme structure through noncovalent tertiary interactions (Figure 1B) [26,27,30,31].

To use the bimolecular form of bacterial RNase P ribozymes as a structural platform to develop ribozyme-based RNA nanostructures, we have developed a chimeric bimolecular ribozyme consisting of the S-domain RNA from the *Thermus thermophilus* (Tth) ribozyme and the C-domain RNA from the *Escherichia coli* (Ec) ribozyme [32]. The resulting chimeric ribozyme exhibited greater stability of the S-domain/C-domain complex than either the bimolecular Tth ribozyme or Ec ribozyme. Based on the chimeric ribozyme, we further prepared a pair of bimolecular ribozymes (Rz-α and Rz-β) in which the RNA–RNA interfaces between the S-domain/C-domain were engineered to achieve orthogonal molecular recognition of the two interfaces (Figure 1B) [32]. Open-form co-oligomers of RNase P ribozyme, in which active Rz-α and Rz-β ribozymes were formed alternately, could be designed by developing unit RNAs (Figure 1C) [30]. For diblock co-oligomer of Rz-α and Rz-β, a pair of unit RNAs were designed by conjugating the domain of Rz-α and the partner domain of the Rz-β. One unit consisted of a structural RNA bearing the S-domain of Rz-α and the C-domain of Rz-β, while the other unit consisted of the S-domain of Rz-β and the C-domain of Rz-α [30]. Each unit was inactive without the partner unit. Through oligomerization of unit RNAs, however, active ribozyme units were formed.

We proposed unit RNAs for open-form oligomers of the RNase P ribozyme (top part of Figure S1, Supplementary Materials). In the initial proposal of unit RNA design, each unit RNA consisted of a pair of RNA strands. Assembly of the pair of RNA strands provided the S-domain and C-domain connected covalently by P12–P3 duplex (top part of Figure S1). The RNA strands in this design, however, had problems of misfolding in their isolated states. Misfolding of the respective RNA strands further prevented the correct assembly of the unit RNAs. We therefore altered the design of the unit module by dissecting the linker duplex of the original design. P12 and P3 were connected noncovalently (bottom part of Figure S1). The noncovalent connection between P12 and P3 duplexes was mediated by a kissing loop (KL) interaction consisting of seven Watson–Crick base pairs.

## 2. Results

### 2.1. Installation of Kissing Loop Elements to Bimolecular RNase P Ribozymes

To oligomerize two bimolecular ribozymes (Rz-α and Rz-β), we selected two distinct KL interactions (x:x′ pair and y:y′ pair) (Figure 1C and Figure S2, Supplementary Materials) [33]. The loop-x and loop-y were introduced into the P12 element in the S-domain RNA, while loop-x′ and loop-y′ were introduced into the P3 element in the C-domain RNA. We first examined the effects of KL elements on the folding of S-domain and C-domain RNAs (Figure S3). In the absence of Mg^2+^ under which conditions the component RNAs dominantly formed secondary structures [34,35,36], the mobilities of both S-domain RNAs and C-domain RNAs on electrophoretic mobility shift assay (EMSA) were similar between Rz-α and Rz-β (Figure S3A). In both S- and C-domain RNAs, the addition of the KL elements retarded their mobilities, the extents of which were also similar between Rz-α and Rz-β (Figure S3A). In the presence of 30 mM Mg^2+^, however, the parent S-domain RNAs of Rz-α (Sα RNA, lane 1 in Figure S3B) and Rz-β (Sβ RNA, lane 7 in Figure S3B) showed distinct mobilities. This result, which has been reported previously [30], reflects distinct tertiary folding of Sα RNA and Sβ RNA in their isolated states.

When the KL elements (loop-x and loop-y) were introduced into the Sα RNA and Sβ RNA, the mobilities of the respective RNAs decreased only slightly (lanes 1–3 and 7–9 in Figure S3B). The identity of the KL element (loop-x or loop-y) scarcely influenced the extents of the changes in mobility. In the presence of 30 mM Mg^2+^, the C-domain RNAs also showed distinct mobilities from the parent Rz-α (αC RNA, lane 4 in Figure S3B) and the parent Rz-β (βC RNA, lane 10 in Figure S3B). The introduction of KL elements (loop-x′ and loop-y′) also decreased the mobilities of the C-domain RNAs (lanes 4–6 and 10–12 in Figure S3B). Although the extents of mobility changes were unaffected by the identity of the KL element, introduction of the x′-loop broadened the band of βC RNA (lane 11 in Figure S3B). While component RNAs (S-RNA and C-RNA) showed distinct mobilities between Rz-α and Rz-β, the bimolecular Rz-α (Sα:αC complex) and Rz-β (Sβ:βC complex) showed virtually the same gel mobilities (Figure S4A), suggesting that the two complexes formed similar tertiary structures.

**Figure 2:**
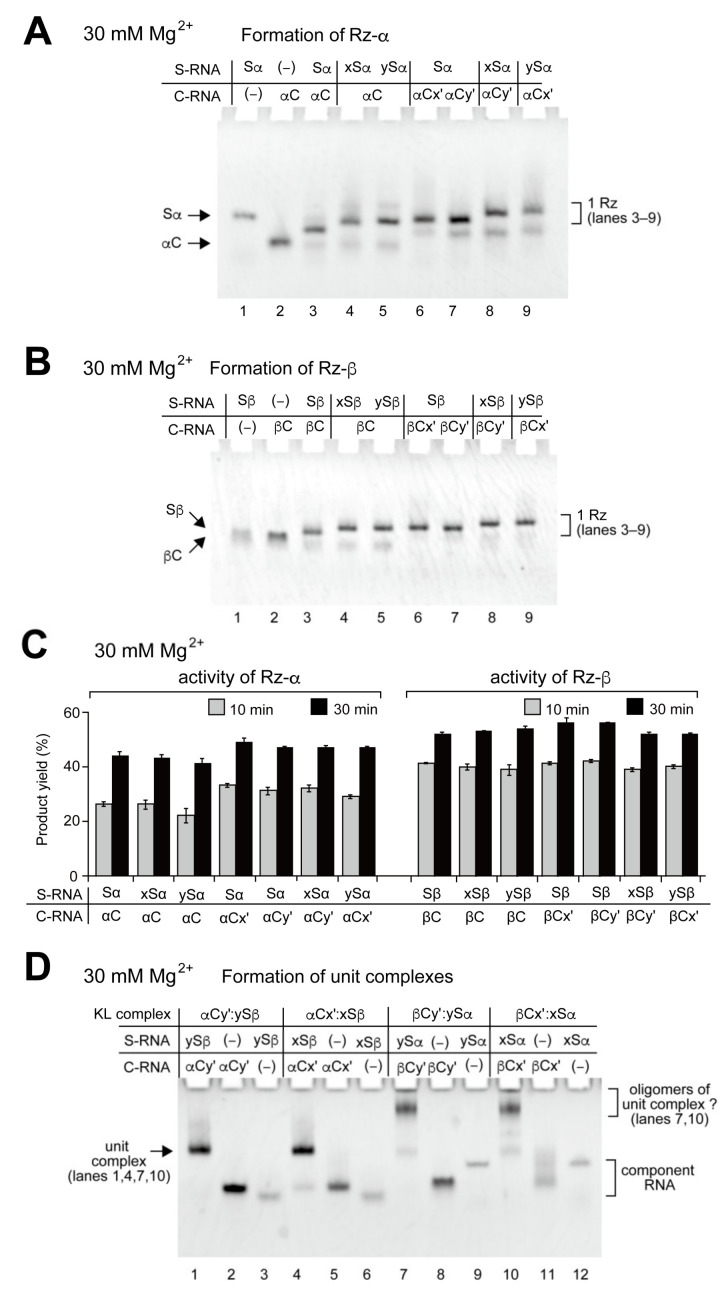
Formation of bimolecular ribozymes and unit complexes. (**A**,**B**) EMSA of Rz-α (**A**) and Rz-β (**B**) bimolecular ribozymes in the presence of 30 mM Mg^2+^. The concentration of each RNA component was 0.5 μM. (**C**) Pre-tRNA cleavage reaction catalyzed by bimolecular ribozymes (Rz-α and Rz-β) in the presence of 30 mM Mg^2+^. Reactions were performed for 10 min and 30 min. The concentration of each RNA component was 0.5 μM. (**D**) EMSA of unit complexes in the presence of 30 mM Mg^2+^. The concentration of each RNA component was 0.5 μM.

We then examined matched assemblies of S-domain RNAs and C-domain RNAs possessing KL elements (Figure 2A,B). To assemble a matched pair of S-domain RNA and C-domain RNA without formation of KL interactions, we paired a given component RNA (S-domain RNA or C-domain RNA) with its partner domain RNA lacking the KL element or with a mismatched KL element (Figure 2A,B). With the matched pair of S- and C-domain RNAs (Sα:αC pair or Sβ:βC pair, lanes 3–9 in Figure 2A,B), each RNA mixture showed a major band, which corresponded to a bimolecular ribozyme (a complex of S-domain RNA and C-domain RNA). The loop-x (or x′) and loop-y (or y′) had nearly the same effects on the mobilities of Rz-α and Rz-β complexes (Figure 2A,B). With mismatched pairs of KLs (x:y′ and y:x′), bimolecular ribozymes were observed as major bands (lanes 8 and 9 in Figure 2A,B). These observations indicated the orthogonality between the x:x′ KL pair and the y:y′ KL pair. To confirm formation of the Rz-α and Rz-β ribozymes, we examined the catalytic activity of each combination of S- and C-domain RNA (Figure 2C). Consistent with the results of EMSA, all matched pairs of the S- and C-domain RNAs exhibited catalytic activities that were comparable to those of the parents, i.e., Rz-α (Sα:αC complex) and Rz-β (Sβ:βC complex) (Figure 2C). These results further support the conclusion that KL elements do not disrupt the catalytic ability of the bimolecular ribozymes.

### 2.2. Formation of Unit Complexes through Kissing Loop Interaction

We next examined the KL interaction-dependent assembly to form an alternative complex of S-domain RNA and C-domain RNA. The resulting bimolecular complexes were designated as a class of unit complexes. With two pairs of KL interactions (x:x′ and y:y′), four distinct unit complexes could be formed depending on the identity of S-domain RNA and C-domain RNA. Two are shown in Figure 1C and the other two are shown in Figure S2B. As each unit complex consists of one S-domain and one C-domain, the total nucleotide length of each unit complex is nearly identical to those of the bimolecular ribozymes. We performed EMSA of the four complexes (Figure 2D). Two complexes (αCy′:ySβ complex and αCx′:xSβ complex) consisted of the S-domain RNA of Rz-β (Sβ RNA) and the C-domain RNA of Rz-α (αC RNA). Each RNA mixture gave a major single band (lanes 1 and 4 in Figure 2D), the mobility of which seemed to be unaffected by the identity of the KL base pairs (y:y′ in lane 1 and x:x′ in lane 4). The other two complexes (βCy′:ySα complex and βCx′:xSα complex) consisted of the S-domain RNA of Rz-α (Sα RNA) and C-domain RNA of Rz-β (βC RNA). While low-mobility bands were formed predominantly (lanes 7 and 10 in Figure 2D), bands of the desired complexes, the mobilities of which should be close to those of the unit complexes of the Sβ RNA and the αC RNA, were faint or absent. The formation of low-mobility bands was independent of the identity of KL interactions (lanes 7 and 10 in Figure 2D). Although the identity of the low-mobility bands and the contribution of KL elements to their formation have not been elucidated, decreasing the Mg^2+^ concentration to 10 mM reduced their relative levels (Figure S4B). These observations suggested that low-mobility bands could be unidentified oligomers containing both Sα RNA and βC RNA. It should also be noted that no complex formation was observed in the previous report between the parent Sα RNA and βC RNA lacking KL elements [30]. The undesired aggregation would be supported by undesired interactions between SαRNA and βC RNA bearing KL elements. Such interactions may be caused by misfolding of the domain RNAs, and the stem regions of KL elements may be involved in the misfolding. The undesired interactions were relatively weaker than the cognate interactions between S-RNA and C-RNA because the undesired aggregation was solved in the formation of the tetramolecular complexes described in the next section.

As the interfaces between the S-domain and C-domain were mismatched in unit complexes, the unit complexes were predicted to be catalytically inactive. Activity assays confirmed this prediction because the four complexes showed no detectable pre-tRNA cleavage activity (Figure S4C). This was consistent with the previous study performed with 50 mM Mg^2+^, under which conditions the mixture of Sβ RNA and αC RNA exhibited no catalytic activity and another mixture (SαRNA and Cβ RNA) had only residual activity [32].

### 2.3. Assembly of Two Ribozymes Mediated by a Kissing Loop Interaction

**Figure 3:**
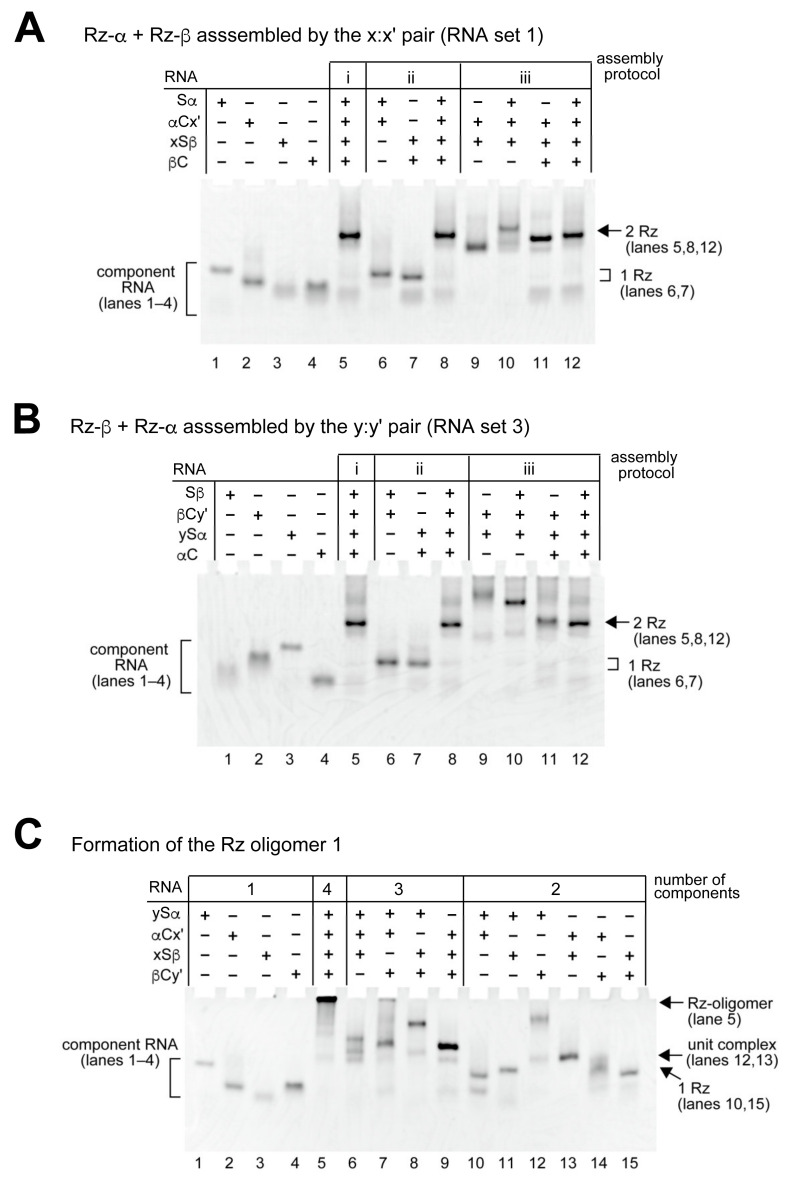
Formation of tetramolecular complexes and ribozyme oligomer containing Rz-α and Rz-β ribozyme units. (**A**) Formation of Rz-α + Rz-β complex mediated by x:x′ KL pair (RNA set 1). The concentration of each RNA component was 1.0 μM. (**B**) Formation of Rz-β + Rz-α complex mediated by y:y′ KL pair (RNA set 3). The concentration of each RNA component was 1.0 μM. (**C**) Formation of alternating co-oligomer consisting of Rz-α and Rz-β (Rz oligomer 1). The concentration of each RNA component was 0.5 μM.

We then examined the assembly of Rz-α (Sα:αC complex) and Rz-β (Sβ:βC complex) through a single KL interaction (x:x′ or y:y′) to form tetramolecular complexes. We first examined the assembly of Rz-α and Rz-β ribozymes by a single KL interaction between αC RNA in Rz-α and Sβ RNA in Rz-β. To assemble the first set of RNA components (RNA set 1: Sα, αCx′, xSβ and βC), we examined three assembly protocols (Figure 3A). The first protocol (protocol i) utilized one-step assembly, in which four RNA components were mixed in a single tube (lane 5 in Figure 3A) to yield a complex (Sα:αCx′:xSβ:βC) directly (lane 5 in Figure 3A). The second and third protocols employed two-step assembly protocols. In the second protocol (protocol ii), Rz-α (Sα:αCx′, lane 6 in Figure 3A) and Rz-β (xSβ:βC, lane 7 in Figure 3A) were assembled separately. The two ribozymes were then mixed to form a matched KL interaction (x:x′) to assemble two ribozymes (Sα:αCx′:xSβ:βC, lane 8 in Figure 3A). In the third protocol (protocol iii), a unit complex (αCx′:xSβ, lane 9 in Figure 3A) was first assembled by a KL interaction. The remaining two RNA components (Sβ and αC) were then added to the RNA solution to form a four-RNA complex (Sα:αCx′:xSβ:βC, lane 12 in Figure 3A). Regardless of the assembly protocol, single major bands were observed in the presence of the four component RNAs (lanes 5, 8, and 12 in Figure 3A).

In protocol iii, assembly of the third component (Sα or βC) to the unit complex (αCx′:xSβ, lane 9 in Figure 3A) was also examined. The addition of Sα RNA or βC RNA afforded a single major band (lanes 10 and 11 in Figure 3A) corresponding to the Sα:αCy′:ySβ complex or the αCx′:xSβ:βC complex, respectively. Essentially the same assembly patterns were also observed with the second set of four RNAs (Sα, αCy′, ySβ, and βC, RNA set 2) (Figure S5A, Supplementary Materials), in which the x:x′ pair used for RNA set 1 was replaced with the y:y′ pair. We then examined the third and fourth sets of RNAs, in which unit KL complexes were formed between βC and Sα. In the case of the unit complex with the y:y′ pair (RNA set 3), assembly of the four-component RNAs afforded a single major band (lanes 5, 8, and 12 in Figure 3B), which corresponded to the RNA tetramer (Sβ:βCy′:ySα:αC) in which Rz-β and Rz-α were formed. Similar assembly patterns were also observed in RNA set 4 (Sβ, βCy′, ySα and αC, Figure S5B), in which the y:y′ pair used for the RNA set 3 was replaced with the x:x′ pair.

### 2.4. Co-Oligomerization of Rz-α and Rz-β by Two KL Interactions

Based on the assembly properties of RNA components through the S/C-domain interfaces and KL interactions, we examined co-oligomerization of two ribozymes (Rz-α and Rz-β) in an alternating manner using two KL interactions (Figure 1C). We comparably designed and examined two co-oligomers of Rz-α and Rz-β, which were termed Rz oligomer 1 (Figure 1C) and Rz oligomer 2 (Figure S2B). In the Rz oligomer 1, the x:x′ KL interaction connected αC RNA and Sβ RNA, whereas the y:y′ KL interaction connected βC RNA and Sα RNA (Figure 1C). In the Rz oligomer 2, the x:x′ KL interaction connected βC RNA and Sα RNA, whereas the y:y′ KL interaction connected αC RNA and Sβ RNA (Figure S2B). In the presence of four RNA components (lane 5 in Figure 3C and Figure S5C), the majority of RNAs remained in the sample well, suggesting that each set of four RNAs formed oligomeric states. In the absence of any one of the four components (lanes 6–9 in Figure 3C and Figure S5C), oligomer bands were resolved except for a residual amount of nonspecific oligomer in lane 7 in Figure 3C and in lane 8 in Figure S5C.

### 2.5. Introduction of Orthogonal Substrate Recognition to Distinguish Activities of Rz-α and Rz-β in the Oligomer

**Figure 4:**
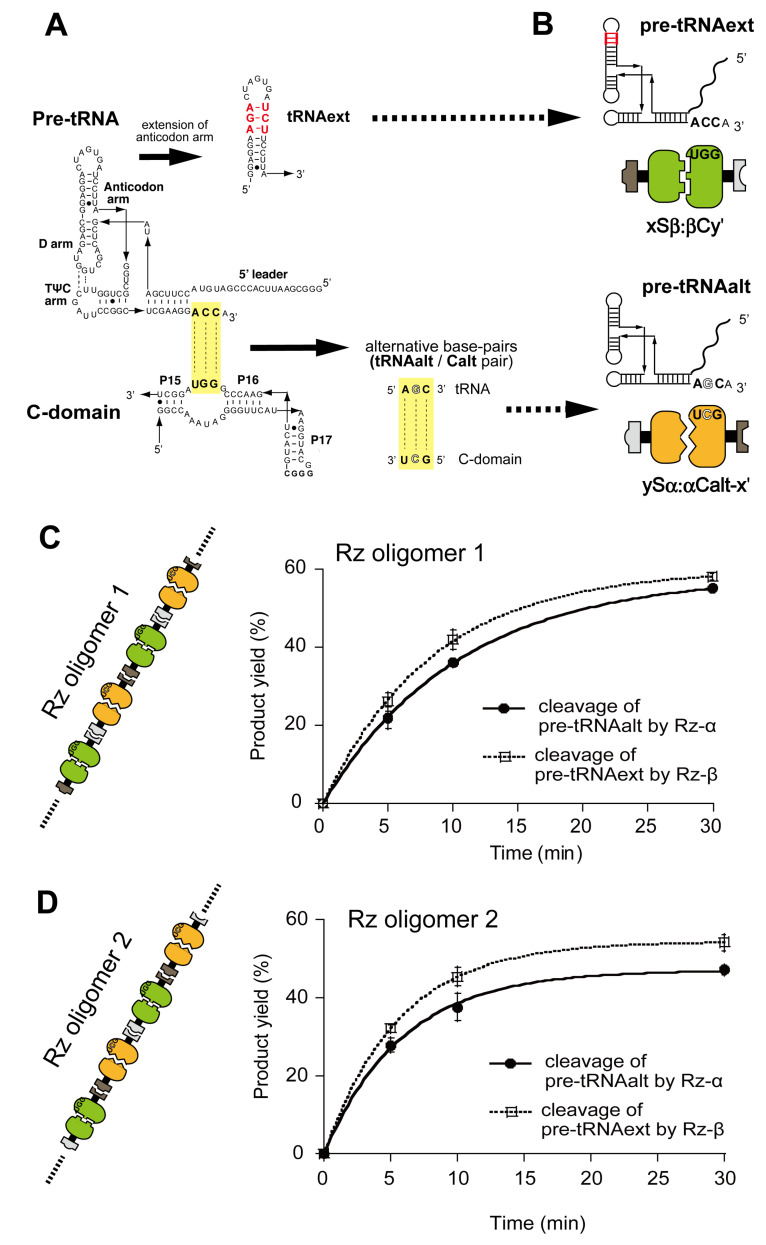
Catalytic activities of Rz-α and Rz-β measured separately in their alternating co-oligomers. (**A**) Engineering to generate an alternative base pair interaction between substrate pre-tRNA and the catalytic domain of RNase P ribozyme and pre-tRNA with an extended anticodon loop. (**B**) Two pairs of pre-tRNA and bimolecular ribozyme. Rz-β was paired with the pre-tRNA with an extended anticodon loop. The alternative base pair interaction was utilized to pair Rz-α and pre-tRNA. (**C**,**D**) Catalytic activities of Rz-α and Rz-β in Rz oligomer 1 (**C**) and Rz oligomer 2 (**D**) in the presence of 30 mM Mg^2+^. Activities of Rz-α and Rz-β were examined with the cleavage of pre-tRNAalt and pre-tRNAext, respectively. The concentration of each RNA component was 0.5 μM.

We then examined the catalytic abilities of Rz oligomers by analyzing the activities of the two ribozymes in the oligomers independently. To distinguish the catalytic activities of Rz-α and Rz-β in the oligomers, it was necessary to differentiate their substrate specificities. We engineered structural elements governing the interaction between RNase P ribozyme and its substrate [37,38,39]. As RNase P ribozymes involving Rz-α and Rz-β recognize pre-tRNAs by their C-domains through three consecutive Watson–Crick base pairs (Figure 4A and Figure S6A, Supplementary Materials), we changed the sequences to generate an altered pair consisting of a variant C-domain RNA (Calt RNA) and its specific pre-tRNA substrate (pre-tRNAalt). We first examined the altered pair using a bimolecular Rz (Sh:hC complex) consisting of Sh RNA and hC RNA (Figure S6B). We produced altRz, composed of Sh RNA and hCalt RNA, to selectively cleave the pre-tRNAalt (Figure S6C). The altRz (Sh:hCalt complex) cleaved the pre-tRNAalt but not the original pre-tRNA (Figure S6D). We also confirmed that the parent ribozyme (Sh:hC complex) poorly cleaved pre-tRNAalt (Figure S6D). We introduced the altered pair to αC RNA and yielded altRz-α (Figure 4B, bottom). To analyze cleavage reactions of the original and altered pre-tRNAs simultaneously, we additionally engineered the original pre-tRNA to alter its nucleotide length. We inserted three base pairs into the anticodon stem of the original pre-tRNA to yield a pre-tRNA variant (pre-tRNAext, Figure 4A). The resulting pre-tRNA variant (pre-tRNAext) was used as a substrate for Rz-β (Figure 4B, top). Substrate and product forms of pre-tRNAext could be separated from those of pre-tRNAalt on polyacrylamide gel electrophoresis (PAGE).

Based on the conditions outlined above, we assayed the activity of Rz oligomer 1 and Rz oligomer 2. In the presence of 30 mM Mg^2+^, with which the component RNAs formed oligomeric forms as determined by EMSA (lane 4 in Figure 3C and Figure S5C), the two substrate pre-tRNAs were both cleaved efficiently (Figure 4B,C, see also lane 2 in Figure S7A,B). When we removed each one of the four components, cleavage of the pre-tRNA matched with the missing component was not observed (Figure S7).

### 2.6. Homo-Oligomerization of a Bimolecular Ribozyme by One Type of Kissing Loop Interaction

Based on the design and characterization of alternating co-oligomers of Rz-α and Rz-β RNAs, we then simplified the oligomer design to produce a homo-oligomer of a bimolecular ribozyme oligomerized by one type of KL interaction.

**Figure 5:**
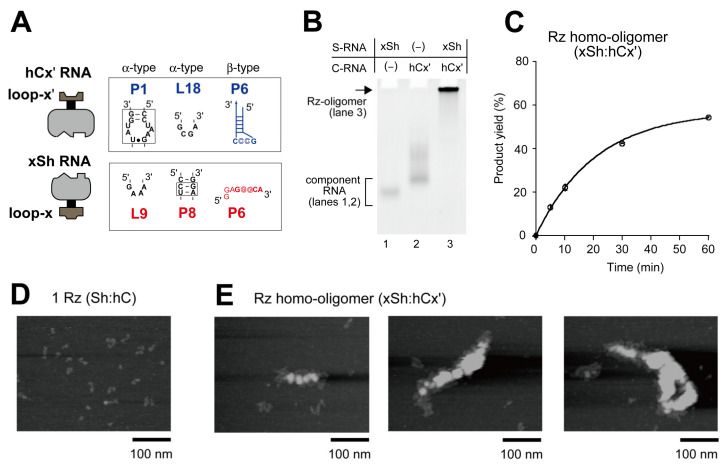
Formation of a ribozyme homo-oligomer. (**A**) Tertiary interactions responsible for the assembly of S-domain and C-domain in a bimolecular ribozyme with the x:x′ KL motif. (**B**) EMSA of the ribozyme homo-oligomer in the presence of 20 mM Mg^2+^. The concentration of each RNA component was 1.0 μM. (**C**) Cleavage of pre-tRNA by the ribozyme homo-oligomer in the presence of 20 mM Mg^2+^. The concentration of each RNA component was 1.0 μM. Some of the error bars are shorter than the size of open circle symbols. (**D**,**E**) AFM imaging of the bimolecular ribozyme (**D**) and the ribozyme homo-oligomers (**E**) in the presence of 20 mM Mg^2+^. The concentration of each RNA component was 1.0 μM.

We employed the bimolecular ribozyme (Sh:hC), to which the x:x′ KL interaction was introduced to design a unit complex (hCx′:xSh) (Figure 5A). The resulting components (xSh RNA and hCx′ RNA) were mixed and analyzed by EMSA in the presence of 20 mM Mg^2+^ (Figure 5B). A high molecular weight band was formed predominantly (Figure 5B), and the sample solution with 20 mM Mg^2+^ also exhibited the ability to cleave the pre-tRNA substrate (Figure 5C).

To further confirm the formation of ribozyme oligomer, we directly examined the sample solution containing xSh RNA and hCx′ RNA by atomic force microscopy (AFM). In the presence of 20 mM Mg^2+^, AFM images of the sample solution containing the components of the parent bimolecular ribozymes (Sh and hC) exhibited dispersed particles corresponding to the Sh:hC complexes (Figure 5D). On the other hand, the sample solution containing components of the homo-oligomer yielded much larger structures (Figure 5E). One image showed an extended structure of length approximately 100 nm (Figure 5E, left). This image resembled those of ribozyme-based oligomers composed of the derivative of the *Tetrahymena* ribozyme [19]. On the other hand, two oligomers were >200 nm in width (Figure 5E, middle and right). These AFM images suggested that these large oligomers may adopt a conformation like a messy and partly overlapping rope. The results of AFM suggested that xSh RNA and hCx′ RNA form oligomers that are large enough that they are unable to migrate on EMSA. Comparison of the activity assay and AFM measurement suggests that oligomerization of RNase P ribozymes into large clusters did not compromise the overall catalytic ability in the buffer solution. Ribozymes buried inside the complex in AFM images can access the pre-tRNA substrates in the solution. In the buffer solution, ribozyme oligomers may form extended shapes and/or exist under the equilibrium of assembly and disassembly occurring rapidly.

## 3. Discussion

In this study, we engineered a bimolecular RNase P ribozyme to construct hetero-oligomers consisting of an orthogonal pair of bimolecular ribozymes (Rz-α and Rz-β). We also installed orthogonality into substrate recognition to distinguish the activities of Rz-α and Rz-β. While the ribozyme oligomers derived from RNase P ribozymes were open-forms in this study, polygonal and three-dimensional nanostructures could be designed and constructed using RNase P ribozymes by following the design strategy for ribozyme-based nanostructures using the *Tetrahymena* group I ribozyme [18,20,21,22]. The development of polygonal-shaped oligomers of RNase P ribozymes is feasible through tuning the KL interacting motifs because these interactions have been used as vertices to assemble RNA duplexes to form RNA oligomers with polygonal shapes [33]. It is also attractive to design ribozyme-based nanostructures using two distinct classes of modular ribozymes (such as RNase P and group I intron), because it would expand the structural and functional diversity of RNA nanostructures. In the case of RNase P and group I intron ribozymes, their catalytic function may be able to cooperate through pre-tRNAs as common substrates. Pre-RNAs, which serve as a major class of substrates for RNase P ribozyme, serve as exon sequences for insertion by group I intron ribozymes [40,41,42]. Some primary transcripts of tRNA molecules possess group I introns to be self-spliced by their ribozyme activity and also the 5′ leader sequence to be cleaved by RNase P ribozyme. As self-splicing group I introns can be rationally converted into spliceosome-type catalysts [43,44], which can excise an intron sequence in a substrate RNA, functional RNA nanostructures that can catalyze the maturation of a pre-tRNA molecule bearing an intron and 5′ leader sequence could be constructed by combining RNase P and group I ribozymes.

To expand structural design of ribozyme-based RNA oligomers and its reliability, it is important to eliminate undesired interactions and assemblies, which were often formed when additional elements were introduced to the parent ribozymes (Figure 2D, lanes 7 and 10) [21,22]. Because they cannot be predicted easily by secondary structure prediction, steady effort may be most effective for optimization of the structural elements to be added. To overcome this problem, experimental and computational approaches to elucidate the structures of undesired interactions and assemblies would be required.

In addition to its importance in RNA nanotechnology, construction of RNase P ribozyme oligomers may contribute to our understanding of naturally occurring RNase P ribozymes and also protein-only RNase P (PRORP) [45], because both types of RNase P have been shown to produce oligomeric forms on crystallization. A bacterial RNase P ribozyme was shown to produce a self-dimeric form in its crystal structure [46] and cryo-electron microscopy (cryoEM) analysis of a minimal PRORP enzyme revealed a screw-like dodecameric assembly [47,48]. Although the functional and evolutionary importance of oligomer formation of RNA-based and protein-based RNase P enzymes are unknown, a constructive approach to RNase P-based RNA nanostructures may provide insights into naturally occurring RNase P enzymes.

## 4. Materials and Methods

### 4.1. Molecular Design

The crystal structure of the *Thermotoga maritima* RNase P ribozyme (PDB ID: 3Q1Q) and the nuclear magnetic resonance (NMR) structure of the RNAI/RNAII inverse KL complex (PDB ID: 2BJ2) were used for construction of 3D models of a ribozyme oligomer (Figure S1) and its components (S-domain RNA and C-domain RNA) possessing KL elements (Figure S2B). Molecular modeling of RNA structures was performed with Discovery Studio (BIOVIA, San Diego, CA, USA).

### 4.2. Plasmid Construction and RNA Preparation

Plasmids encoding sequences of S-domains and C-domains of bimolecular ribozymes (Rz-α, Rz-β, and Ch:hS) were described previously [32]. Introduction of KL elements into the above plasmids and extension of the pre-tRNA anticodon stem were achieved by site-directed mutagenesis using inverse-PCR. Plasmids were then used as templates for PCR to amplify DNA fragments for use as templates for in vitro transcription. The substrate pre-tRNA (human tyrosyl pre-tRNA) was transcribed using a DNA fragment encoding human tyrosyl pre-tRNA as a template, which was prepared by PCR using the plasmid pHSTY [49] as the template. The T7 promoter sequence was added by PCR with a sense primer containing the promoter sequence. For preparation of DNAs with altered base pairs between the pre-tRNA and the C-domain RNA of Rz-α, a template DNA for pre-tRNAalt was amplified by PCR using an antisense primer containing the complementary sequence to the alternative base pairs in its 5′ region. A template DNA for the C-domain RNA with alternative base pairs was amplified by PCR using a sense primer containing the sequence of the alternative base pairs. Transcription reactions with T7 RNA polymerase were performed at 37 °C for 4.5 h in 50 mM Tris-HCl (pH 7.5) buffer solution containing Mg^2+^ (15 mM) and nucleoside triphosphates (1 mM each). The DNA template in the solution was removed by treatment with DNase I for 30 min. Transcribed RNA was then purified by 9% denaturing PAGE. The purified pre-RNA and its variants were then labeled with boron dipyrromethene (BODIPY) fluorophore [50].

### 4.3. Gel Electrophoresis Mobility Shift Assay (EMSA)

An aqueous solution containing components of a bimolecular ribozyme, unit complex, or ribozyme oligomers (the final concentrations of each component are indicated in the legend of each figure) was heated at 85 °C for 5 min. To this solution was added 10× concentrated folding buffer (final concentrations: 80 mM Tris-acetate, pH 7.5, and 20 mM or 30 mM Mg(OAc)_2_). The resulting mixture was incubated at 37 °C for 30 min and then at 4 °C for an additional 30 min. In the case of two-step assembly using in formation of Rz oligomers, RNA solutions prepared in step 1 were incubated at 37 °C for 30 min, mixed in step 2, and additionally incubated at 37 °C for 30 min followed by at incubation at 4 °C for an additional 30 min. After addition of 6× loading buffer containing 50% glycerol and 0.1% xylene cyanol, the samples were loaded onto a 5% nondenaturing polyacrylamide gel (29:1 acrylamide:bisacrylamide) containing 80 mM Tris-acetate (pH 7.5) and 20 mM or 30 mM Mg(OAc)_2_. Electrophoresis was carried out at 4 °C, 200 V for the initial 5 min, followed by 75 V for 5 h. RNAs were visualized by staining of gels with ethidium bromide after electrophoresis.

### 4.4. Ribozyme-Catalyzed Pre-tRNA Cleavage Reactions

An aqueous solution of pre-tRNA and an aqueous solution containing components of a bimolecular ribozyme, unit complex, or ribozyme oligomers were prepared separately. These solutions were heated at 85 °C for 5 min. A 3× concentrated reaction buffer (final concentrations: 50 mM Tris-HCl, 20 mM or 30 mM MgCl_2_) was added to each solution. The resulting solutions were further incubated at 37 °C for 30 min to fold pre-tRNA or assemble bimolecular ribozyme, unit complex, or ribozyme oligomers. The two solutions were then mixed to initiate the substrate cleavage reaction catalyzed by RNase P ribozymes. Aliquots were taken at given time points and treated with 1.0 volume of stop solution containing 75% formamide, 0.1% xylene cyanol, and 100 mM EDTA. The reaction mixtures were separated by electrophoresis in 9% (*w/v*) polyacrylamide gels containing 8 M urea. The substrate and reaction products were analyzed using a Pharos FX fluoroimager (BioRad, Hercules, CA, USA). All of the ribozyme activity assays were repeated at least twice, and the mean values are shown in the figures with the minimum and maximum values indicated by error bars.

### 4.5. Atomic Force Microscopy (AFM)

AFM was performed with a high-speed instrument (Nano Live Vision; RIBM, Tsukuba, Japan). Assembly of RNA components in buffer (final concentration: 80 mM Tris-acetate (pH 7.5), 20 mM Mg(OAc)_2_) was performed according to the protocol for EMSA. Concentrations of RNAs in the solution were then adjusted to ~80 nM by addition of sample loading buffer (80 mM Tris-acetate, 20 mM Mg(OAc)_2_, pH 7.5). The resulting sample (2 μL) was deposited onto a mica surface coated with 0.1% (3-aminopropyl)triethoxysilane. After incubation for 5 min, the mica surface was washed gently with sample loading buffer. The samples in the sample loading buffer solution were imaged at room temperature. A silicon nitride cantilever (BL-AC10EGS; Olympus Corporation, Tokyo, Japan) with a spring constant of 0.1–0.2 N/m and a resonant frequency of 400–1000 kHz was used. AFM imaging was carried out at a scan rate of 0.2 fps.

## 5. Conclusions

We engineered a bimolecular RNase P ribozyme to construct its open-form oligomers, in which ribozyme units were connected by KL interactions. The resulting ribozyme oligomers were investigated by EMSA and AFM, which provided experimental data supporting oligomerization of the unit RNAs. Although this is the initial step toward modular structural engineering of RNase P ribozymes, this study expanded the repertoire of structural platforms of modular ribozymes for RNA nanotechnology.

## Data Availability

Not applicable.

## Supplementary Materials

The following supporting information can be downloaded at: https://www.mdpi.com/article/10.3390/molecules27238298/s1, Figure S1: molecular design of open-chain ribozyme assemblies, in which bimolecular RNase P ribozymes serve as functional units with catalytic activity; Figure S2: secondary structures of bimolecular RNase P ribozymes and their open-chain assembly; Figure S3: native gel electrophoresis of component RNAs; Figure S4: formation of bimolecular ribozymes and bimolecular unit complexes; Figure S5: formation of tetramolecular complexes and a ribozyme oligomer containing Rz-α and Rz-β ribozyme units; Figure S6: alternative base pair interaction to assemble substrate pre-tRNA and the ribozyme; Figure S7: characterization of Rz-α and Rz-β ribozyme units formed in ribozyme co-oligomers.
