# Supplementary material for: Catalytic RNA Oligomers Formed by Co-Oligomerization of a Pair of Bimolecular RNase P Ribozymes
Source: Molecules. 2022 Nov 28;27(23):8298. doi: 10.3390/molecules27238298 (PMC9740620; doi:10.3390/molecules27238298)
Supplement: Supplementary file 1 [file molecules-27-08298-s001.zip › molecules-2028077-supplementary.pdf]

Supplementary Materials

For

Catalytic RNA Oligomers Formed by Co-Oligomerization of a Pair of Bimolecular RNase P Ribozymes

Mst. Ayesha Siddika, Takahiro Yamada, Risako Aoyama, Kumi Hidaka,

Hiroshi Sugiyama, Masayuki Endo, Shigeyoshi Matsumura, Yoshiya Ikawa

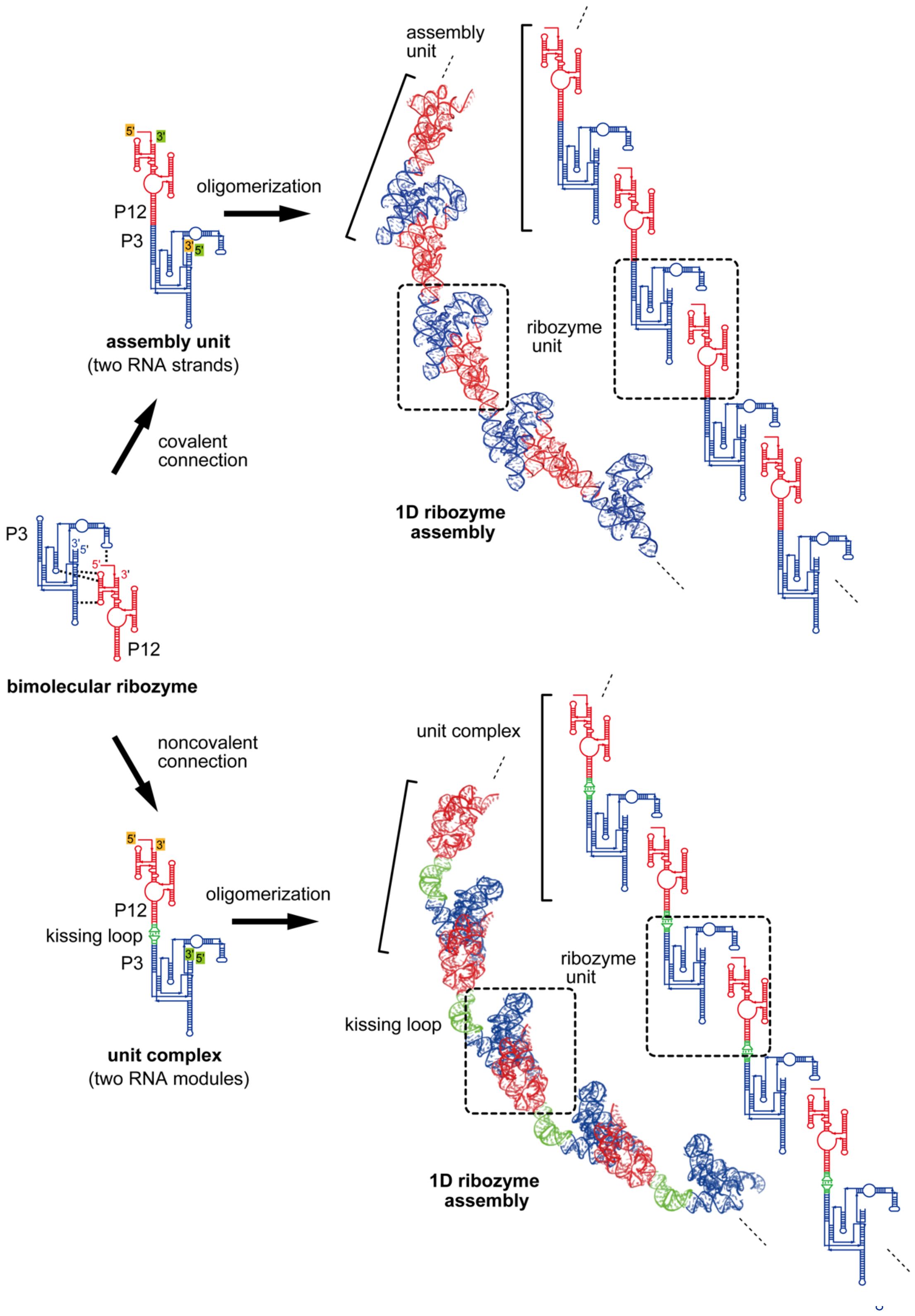

Figure S1
Molecular design of open-chain ribozyme assemblies, in which bimolecular RNase P ribozymes serve as functional units with catalytic activity. In the previous design shown at the top, the P12 element of the S-domain RNA (red) and the P3 element of the C-domain RNA (blue) are connected covalently. In the design used in this study shown at the bottom, the P12 element and the P3 element are connected noncovalently.

A

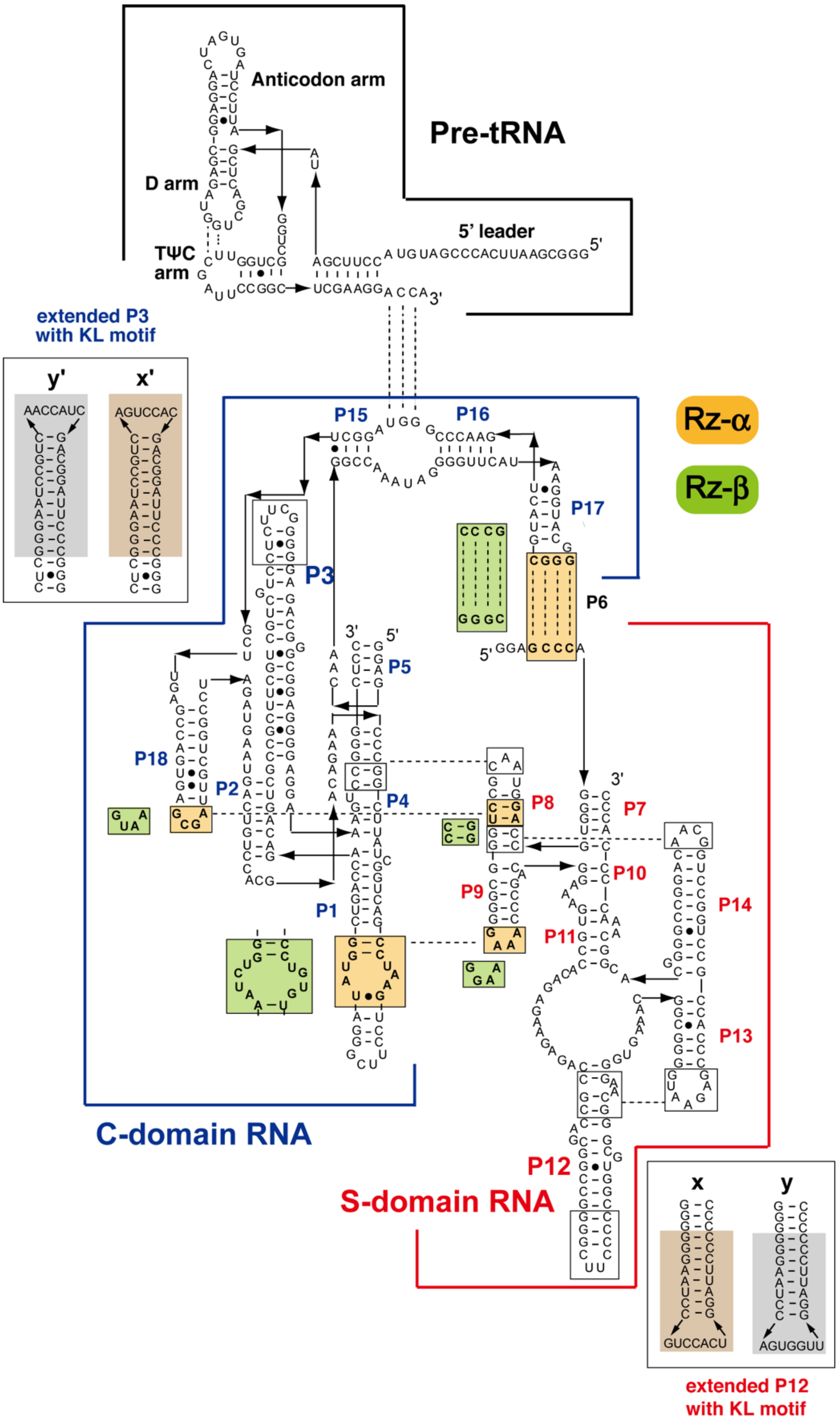
Pre-tRNA
Anticodon arm
D arm
TΨC arm
5' leader
extended P3 with KL motif
y'
x'
Rz-α
Rz-β
P15
P16
P17
P3
P6
P5
P18
P2
P4
P8
P7
P1
P9
P10
P14
P11
P13
P12
C-domain RNA
S-domain RNA
x
y
extended P12 with KL motif

B

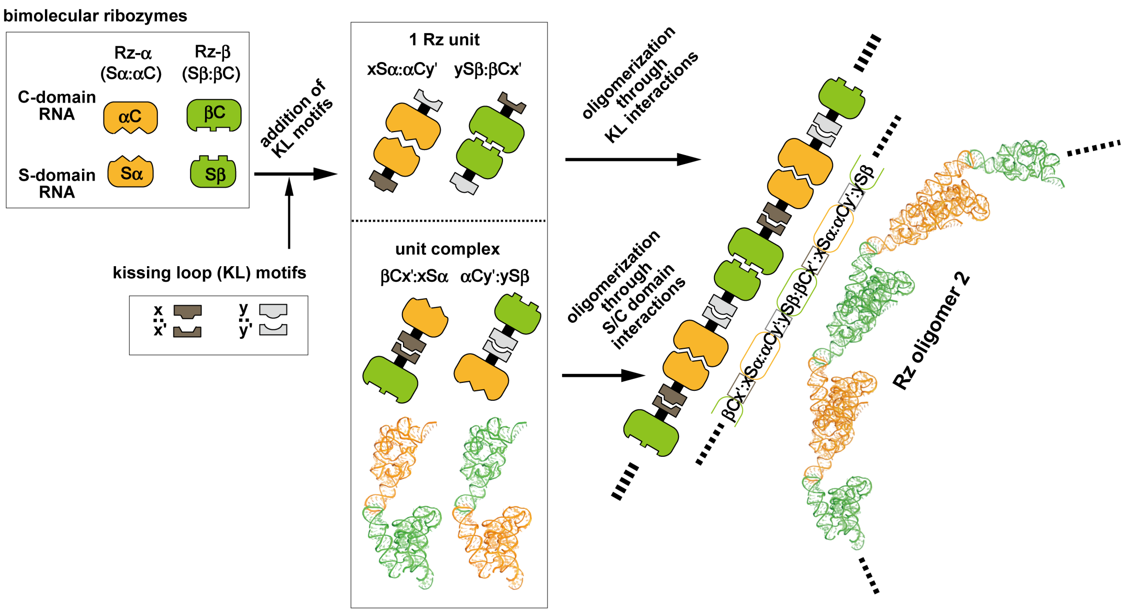

Figure S2

Secondary structures of bimolecular RNase P ribozymes and their open-chain assembly.

A) Nucleotide sequences and secondary structures of Rz-α and Rz-β bimolecular ribozymes. Three RNA motifs are different between Rz-α and Rz-β. RNA motifs used in Rz-α and Rz-β are shown in orange and green, respectively. The element responsible for interaction with pre-tRNA is also shown. In derivatives of the S-domain and C-domain RNAs for oligomer formation, P12 and P3 elements shown as solid boxes were replaced with extended stem loops possessing KL motifs.

B) Scheme of modular engineering to generate bimolecular RNase P ribozymes possessing KL motifs enabling co-oligomerization to yield Rz oligomer 2.

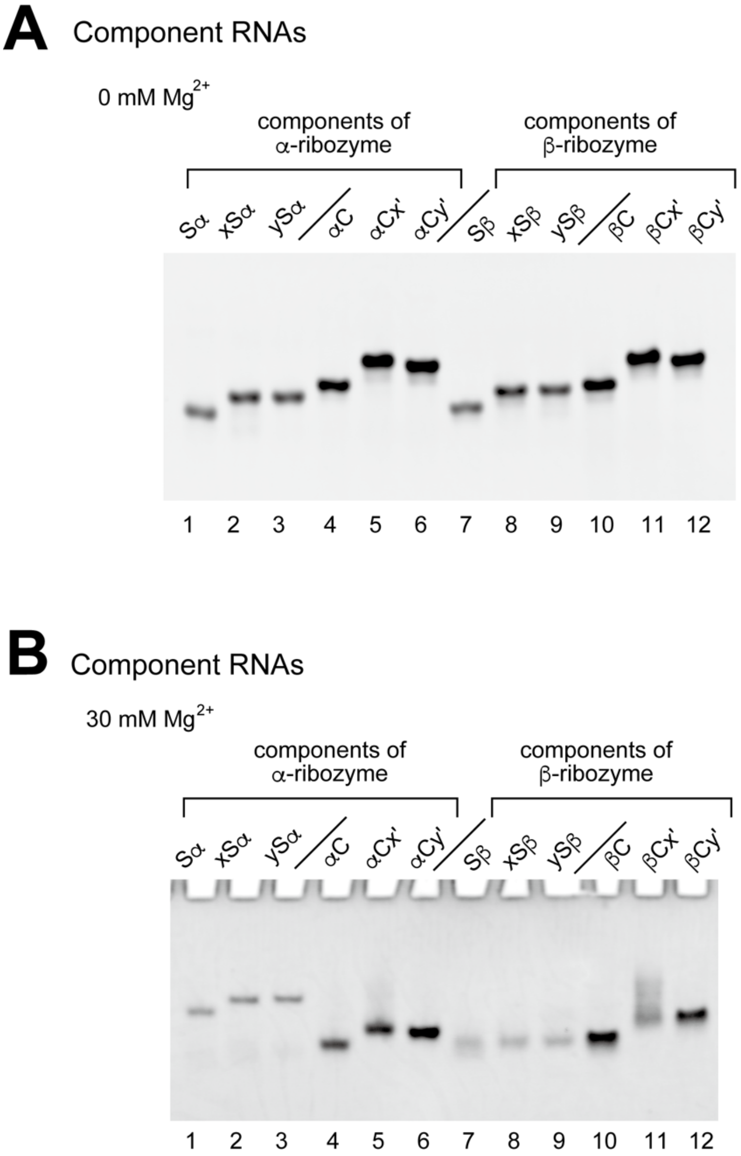

Figure S3
Native gel electrophoresis of component RNAs
A) Native gel electrophoresis of component RNAs in the absence of Mg^{2+}. The concentration of each RNA component was 0.5 μM.
B) Native gel electrophoresis of component RNAs in the presence of 30 mM Mg^{2+}. The concentration of each RNA component was 0.5 μM.

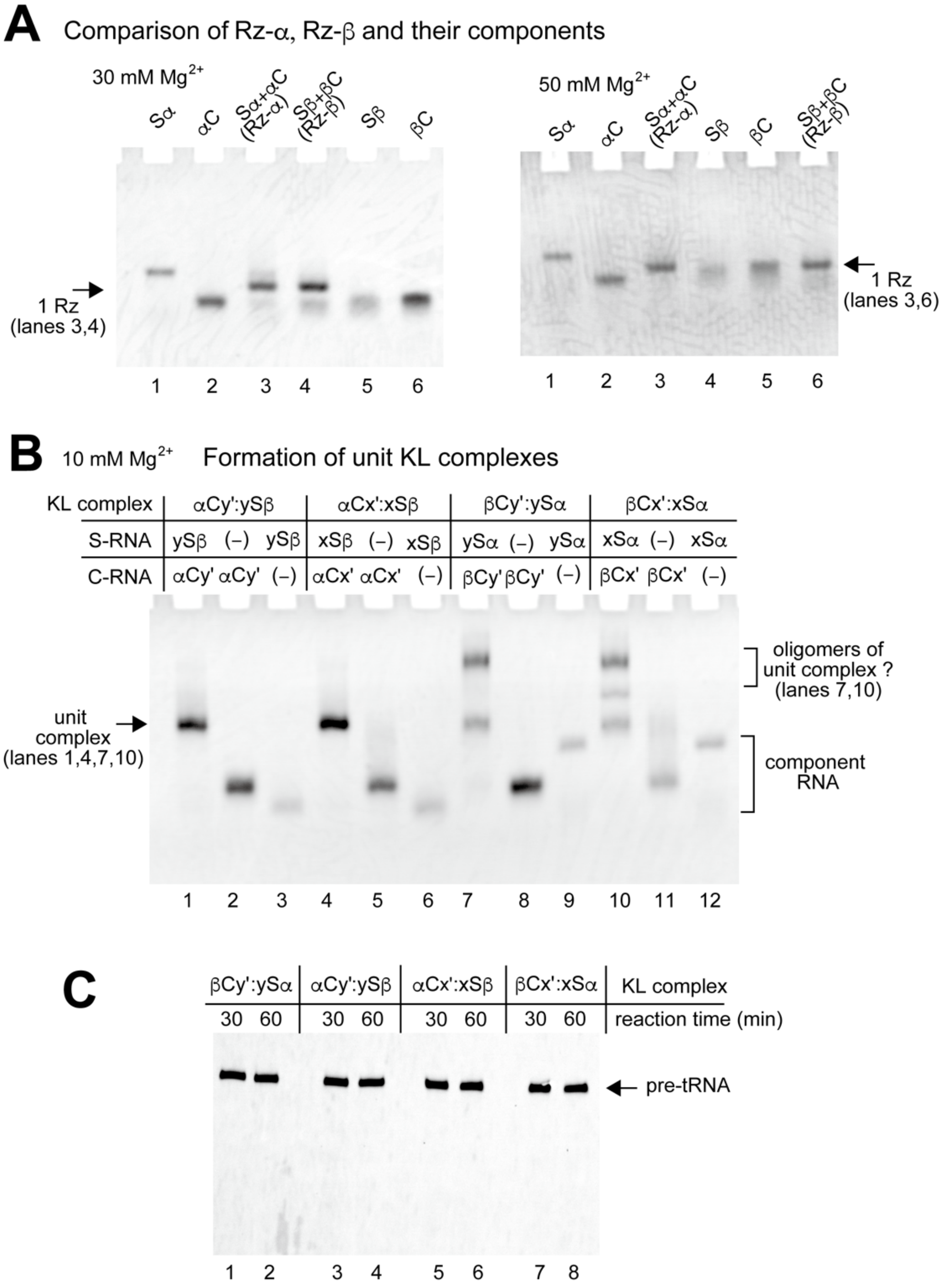

Figure S4

Formation of bimolecular ribozymes and bimolecular unit complexes.

A) EMSA of Rz-α and Rz-β bimolecular ribozymes and their components in the presence of 30 mM (left) and 50 mM (right) Mg^{2+}. The concentration of each RNA component was 0.5 μM.

B) EMSA of unit complexes in the presence of 10 mM Mg^{2+}. The concentration of each RNA component was 0.5 μM.

C) Pre-tRNA cleavage reaction assay with unit complexes in the presence of 30 mM Mg^{2+}. Reactions were performed for 30 min and 60 min. The concentration of each RNA component was 0.5 μM.

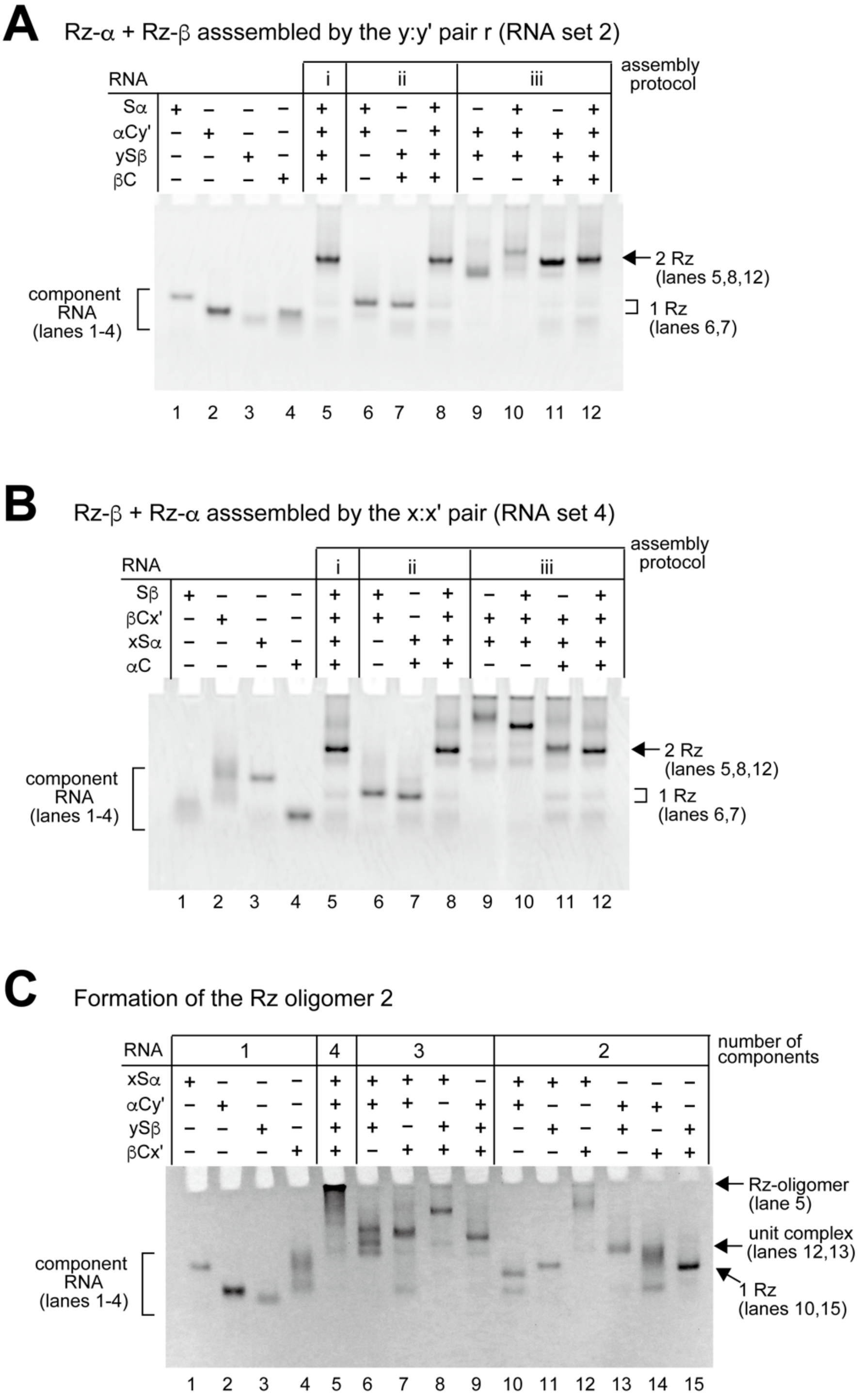

Figure S5

Formation of tetramolecular complexes and a ribozyme oligomer containing Rz-α and Rz-β ribozyme units.

A) Formation of Rz-α + Rz-β complex mediated by y:y′ KL pair (RNA set 2). The concentration of each RNA component was 1.0 μM.

B) Formation of Rz-β + Rz-α complex mediated by x:x′ KL pair (RNA set 4). The concentration of each RNA component was 1.0 μM.

C) Formation of alternating co-oligomer consisting of Rz-α and Rz-β (Rz oligomer 2). The concentration of each RNA component was 0.5 μM.

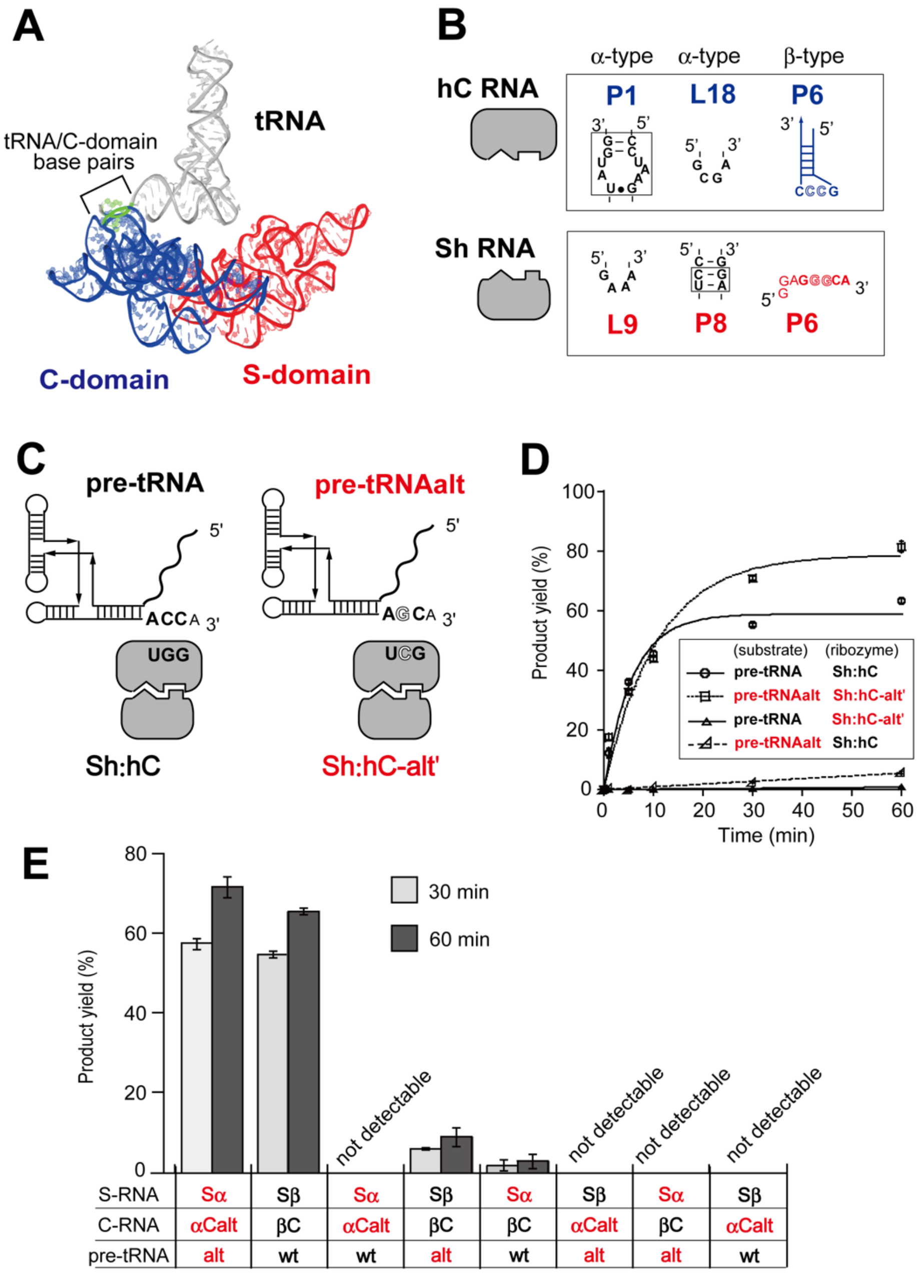

Figure S6

Alternative base pair interaction to assemble substrate pre-tRNA and the ribozyme.

A) Crystal structure of a complex of the RNase P ribozyme from *Thermotoga maritima* and tRNA. Base pairs between tRNA and the C-domain is shown in green.

B) Tertiary interactions assembling the C-domain and S-domain in a bimolecular ribozyme used for characterization of the alternative base pairs.

C) The parent (left) and alternative (right) base pairs between the C-domain of the bimolecular ribozyme and pre-tRNA.

D) Cleavage of pre-tRNAs possessing the parent and alternative base pair element by the ribozyme (Sh:hC) with the parent C-domain RNA (hC) and its variant (hC-alt′) possessing the alternative base pair element. The concentration of each RNA component was 0.5 μM. Some of the error bars are shorter than the size of the symbols.

E) Cleavage of pre-tRNAs possessing the parent and alternative base pair element by Rz-β (Sβ:βC) and a variant Rz-α (Sα:αCalt) possessing the alternative base pair element. The concentration of each RNA component was 0.5 μM.

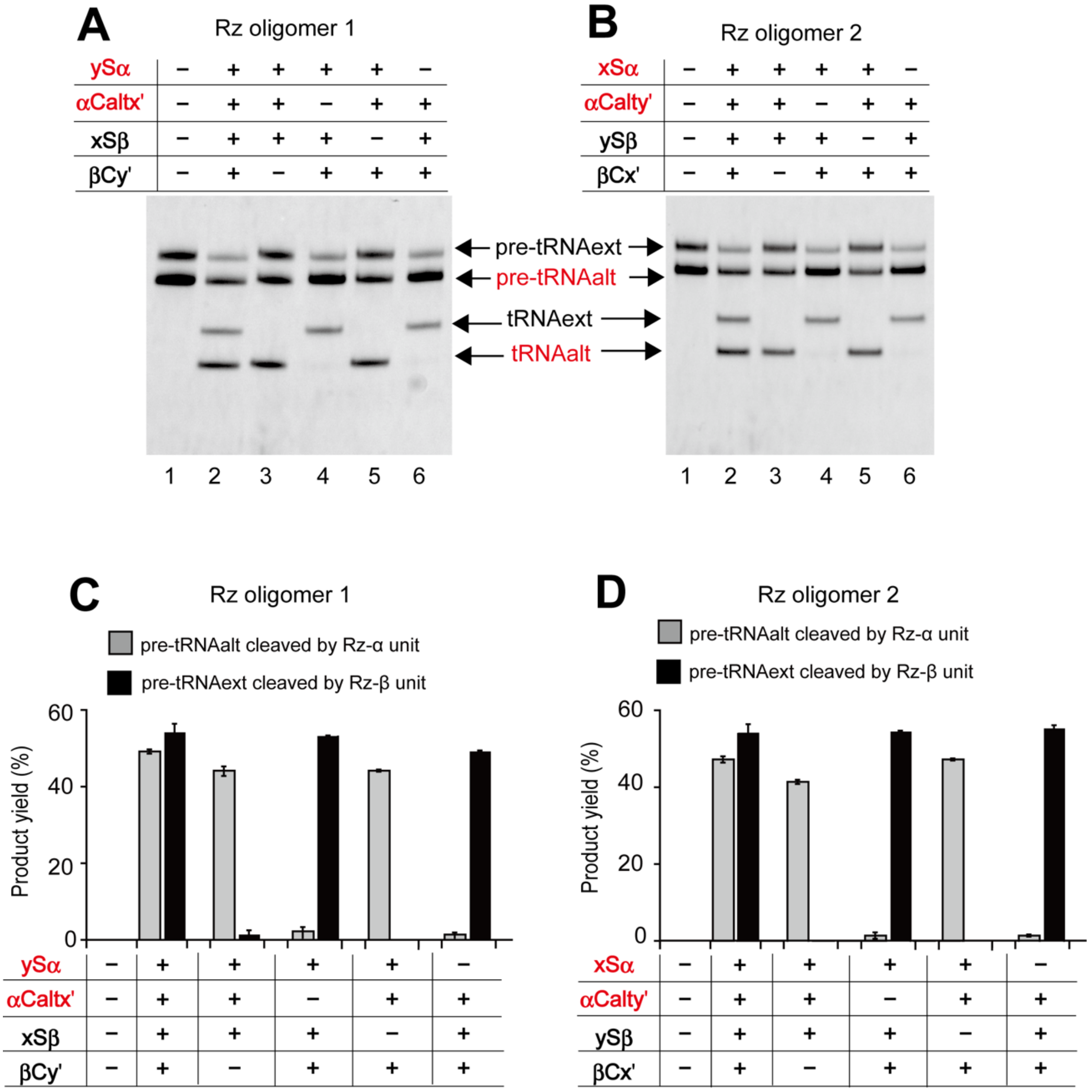

Figure S7

Characterization of Rz-α and Rz-β ribozyme units formed in ribozyme co-oligomers.

A, B) Cleavage of pre-tRNAs possessing the parent and alternative base pair element by Rz oligomer 1 (A) and Rz oligomer 2 (B), which both consisted of Rz-β (Sβ:βC) and a variant Rz-α (Sα:αCalt). The concentration of each RNA component was 0.5 μM.

C, D) Extent of pre-tRNA cleavage reactions catalyzed by Rz oligomer 1 (C) and Rz oligomer 2 (D). The concentration of each RNA component was 0.5 μM.
